# South-East Asian strains of *Plasmodium falciparum* display higher ratio of non-synonymous to synonymous polymorphisms compared to African strains

**DOI:** 10.12688/f1000research.9372.2

**Published:** 2016-10-21

**Authors:** Gajinder Pal Singh, Amit Sharma

**Affiliations:** 1 Molecular Medicine Group, International Centre for Genetic Engineering and Biotechnology (ICGEB), New Delhi, India

**Keywords:** artemisinin resistance, resistance evolution, Plasmodium falciparum, non-synonymous polymorphism

## Abstract

Resistance to frontline anti-malarial drugs, including artemisinin, has repeatedly arisen in South-East Asia, but the reasons for this are not understood. Here we test whether evolutionary constraints on *Plasmodium falciparum *strains from South-East Asia differ from African strains. We find a significantly higher ratio of non-synonymous to synonymous polymorphisms in *P. falciparum *from South-East Asia compared to Africa, suggesting differences in the selective constraints on *P. falciparum *genome in these geographical regions. Furthermore, South-East Asian strains showed a higher proportion of non-synonymous polymorphism at conserved positions, suggesting reduced negative selection. There was a lower rate of mixed infection by multiple genotypes in samples from South-East Asia compared to Africa. We propose that a lower mixed infection rate in South-East Asia reduces intra-host competition between the parasite clones, reducing the efficiency of natural selection. This might increase the probability of fixation of fitness-reducing mutations including drug resistant ones.

## Introduction

Artemisinin combination therapy (ACT) is the frontline treatment for malaria caused by *Plasmodium falciparum* and has played a major role in reducing malaria mortality from an estimated 840,000 deaths in the year 2000 to 440,000 deaths in the year 2015 ^1^. The emergence and spread of artemisinin resistance in South-East Asia, however, poses a serious threat to malaria control, and the containment of artemisinin resistance is thus a global public heath priority ^2– 8^.

One of the most important unanswered questions in anti-malarial drug resistance is why it has repeatedly emerged in South-East Asia ^3, 5, 6, 9^. The resistance to chloroquine was first reported in South-East Asia in 1957 before spreading to India and Africa where it resulted in the significant increase in malaria child mortality possibly killing millions of children ^10– 12^. The resistance to sulphadoxine-pyrimethamine also emerged in South-East Asia in the late 1960s following a similar route to India and Africa ^9^. Worryingly, the resistance to artemisinin has emerged independently at multiple places in South-East Asia ^13– 17^ and is now present 25 km from the Indian border ^16^ threatening to follow the same trajectory as resistance to previous anti-malarial drugs. Improved understanding of the process of how and why anti-malarial drug resistance emerges in South-East Asia could provide critical information in developing strategies to prevent the spread of the current wave of artemisinin resistance.

Here we ask whether there are evolutionary constraints on *P. falciparum* strains from South-East Asia that differ from African strains and thus might explain the higher predisposition of South-East Asia strains to evolve drug resistance. To address this question we utilized a recent large global genome sequencing data from ~3400 clinical samples which identified nearly million high-quality single nucleotide polymorphisms (SNPs) in the exonic regions of *P. falciparum* ^18^.

## Results

### Higher ratio of non-synonymous to synonymous polymorphism in *P. falciparum* from South-East Asia

**Figure 1.**
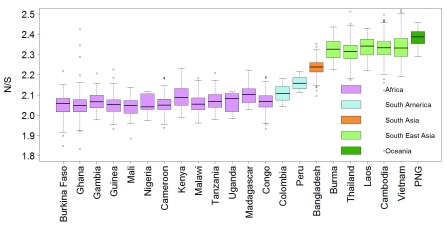
Higher ratio of non-synonymous to synonymous polymorphism in *P. falciparum* samples from SEA. The ratios of non-synonymous to synonymous polymorphism (N/S) for 3394 samples from 22 countries are shown. The y-axis is truncated at the top with 13 samples not shown. PNG - Papua New Guinea.

**Figure 2.**
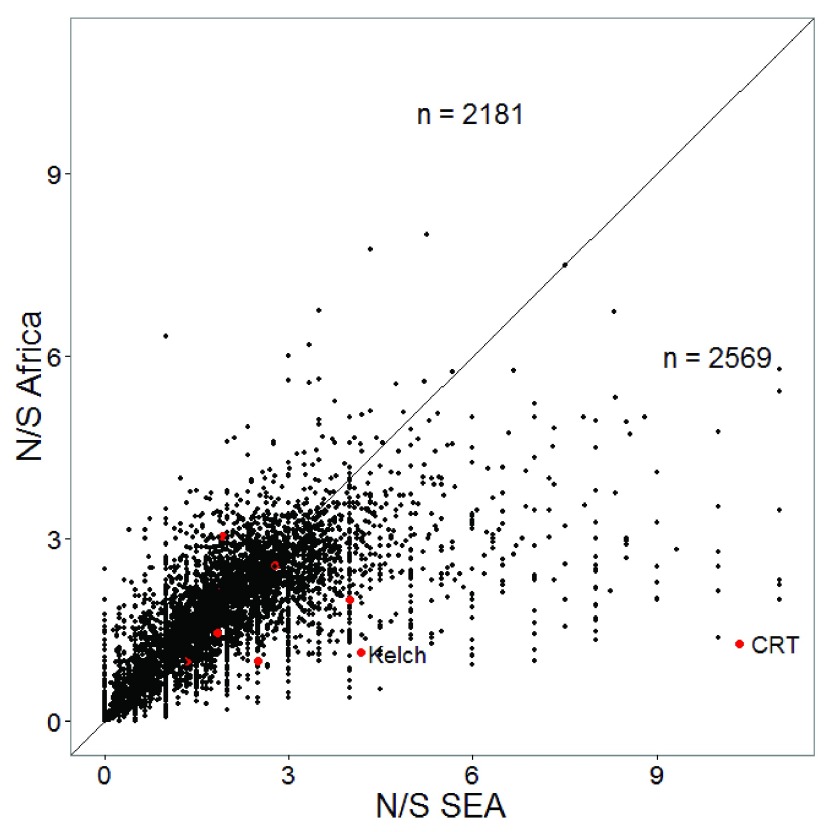
*P. falciparum* genes show a higher rate N/S in SEA compared to Africa. The scatter-plot shows N/S in SEA and Africa for 4792 genes. Genes previously associated with artemisinin resistance in a GWAS study ^14^ are shown in red, with Kelch13 and chloroquine-resistance transporter (CRT) labelled. The diagonal line is shown and the numbers of genes on both sides of the diagonal are indicated. The x and y-axes are truncated with 28 genes not shown.

Resistance to anti-malarial drugs often involves changes in the amino-acid sequence within specific proteins. Thus, we tested whether the ratio of non-synonymous (amino acid changing) to synonymous polymorphism is higher in South-East Asia (SEA). Figure 1 shows a significantly higher ratio of non-synonymous to synonymous polymorphism (N/S) in SEA samples compared to African samples with almost no overlap in their distributions. The mean and median N/S for samples from SEA were 2.33, compared to 2.06 for Africa (Wilcox test p-value 0, number SEA samples 1600, and number Africa samples 1647). The higher N/S in SEA compared to Africa was also evident at the gene level with a larger number of genes showing higher N/S in SEA than in Africa ( Figure 2). Mean and median N/S for genes in SEA samples were 2.1 and 1.9 respectively, while for African samples the mean and median N/S were 1.9 and 1.8 respectively (paired t-test p-value 1E-43, paired Wilcox-test p-value 4E-27, n = 4792). There were 75 genes with more than 3-fold higher N/S in SEA samples relative to African samples and N/S of more than four in SEA. Interestingly, most of these genes were not related to antigenic variation ( Supplementary Table 1), but perform basic housekeeping functions, suggesting that higher N/S of these genes in SEA might not be primarily driven by differential host immune selection. In addition to *kelch13*, -the only gene known to be causally associated with artemisinin resistance- the list includes CRT (chloroquine-resistance transporter) which shows an 8-fold higher N/S in SEA samples compared to African samples and has previously been shown to be associated with artemisinin resistance in a genome-wide association studies (GWAS) study ^14^. In summary, *P. falciparum* strains from SEA show a higher ratio of non-synonymous to synonymous polymorphisms than African strains.

### Higher non-synonymous changes at the conserved positions in South-East Asia

**Figure 3.**
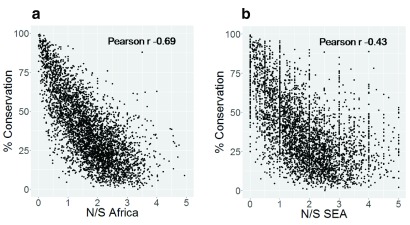
Lower correlation between N/S and protein conservation in SEA. **A**) Scatter-plot of N/S in Africa and percent protein conservation and **B**) Scatter-plot of N/S in SEA and percent protein conservation. Percent conservation for each protein is the percent of residues identical across orthologs in seven *Plasmodium* species ( *P. berghei, P. chabaudi, P. cynomolgi, P. knowlesi, P. reichenowi, P. vivax, P. yoelii)*. Only proteins with orthologs in all *Plasmodium* species are shown (4075 proteins). Y-axis is truncated with 7 points not shown in Figure 3a and 112 points not shown in Figure 3b.

**Figure 4.**
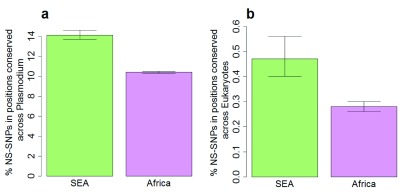
Non-synonymous polymorphisms specifically observed in SEA are more likely to occur at conserved positions when compared to non-synonymous polymorphisms specifically observed in Africa. **A**) Conserved sites were defined as sites identical across orthologs in *Plasmodium* species ( *P. berghei, P. chabaudi, P. cynomolgi, P. knowlesi, P. reichenowi, P. vivax, P. yoelii*) in multiple sequence alignment. **B**) Conserved sites were defined as sites identical across orthologs in diverse eukaryotes ( *S. cerevisiae*, *D. melanogaster*, *C. elegans, H. sapiens*) in multiple sequence alignment. Error bars indicate 95% confidence intervals of the mean from 1,000 bootstrap samples.

**Figure 5.**
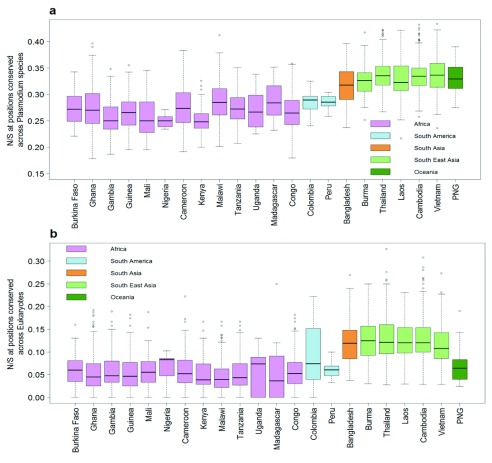
Higher ratio of non-synonymous to synonymous polymorphism at conserved sites in *P. falciparum* from SEA. **A**) Box-plot showing N/S for 3394 samples from 22 countries at sites identical across orthologs in seven *Plasmodium* species in multiple sequence alignment. **B**) Box-plot showing the N/S ratio at sites identical across orthologs in diverse eukaryotes ( *S. cerevisiae*, *D. melanogaster*, *C. elegans* and *H. sapiens*) in multiple sequence alignment. The y-axis is truncated at the top with 10 samples not shown in both panels. PNG - Papua New Guinea.

Highly conserved proteins in *P. falciparum* show a much lower N/S, indicating the lower tolerance for non-synonymous polymorphism ^18^. We tested whether the correlation between N/S and protein conservation might be different in SEA and Africa. The correlation between N/S and conservation was much weaker in SEA ( Figure 3) with Pearson correlation of -0.43 (95% CI: -0.41 to -0.46) compared to -0.69 (95% CI: -0.68 to -0.71) in Africa. The lower correlation in SEA suggests a higher ratio of non-synonymous to synonymous changes at conserved positions. Indeed, non-synonymous polymorphisms specifically observed in SEA are more likely to occur at conserved positions compared to those specific to Africa ( Figure 4). Samples from SEA show higher N/S compared to Africa when considering only conserved positions ( Figure 5). These results suggest a lower efficiency of negative selection in SEA in removing potentially deleterious mutations. This may be important for the acquisition of antimalarial drug resistance since drug-resistance mutations preferentially occur at the conserved sites ^19^, *e.g.* artemisinin resistance mutations in Kelch13 occur in the conserved region of the protein ^18^, resistance mutations also occur in the conserved regions in DHFR (dihydrofolate reductase), DHPS (dihydropteroate synthase), and CRT (chloroquine-resistance transporter) ^19^. In summary, *P. falciparum* strains from SEA show a higher ratio of non-synonymous to synonymous polymorphisms at conserved sites in the protein sequences than African strains.

### Lower mixed infection rate in South-East Asia

**Figure 6.**
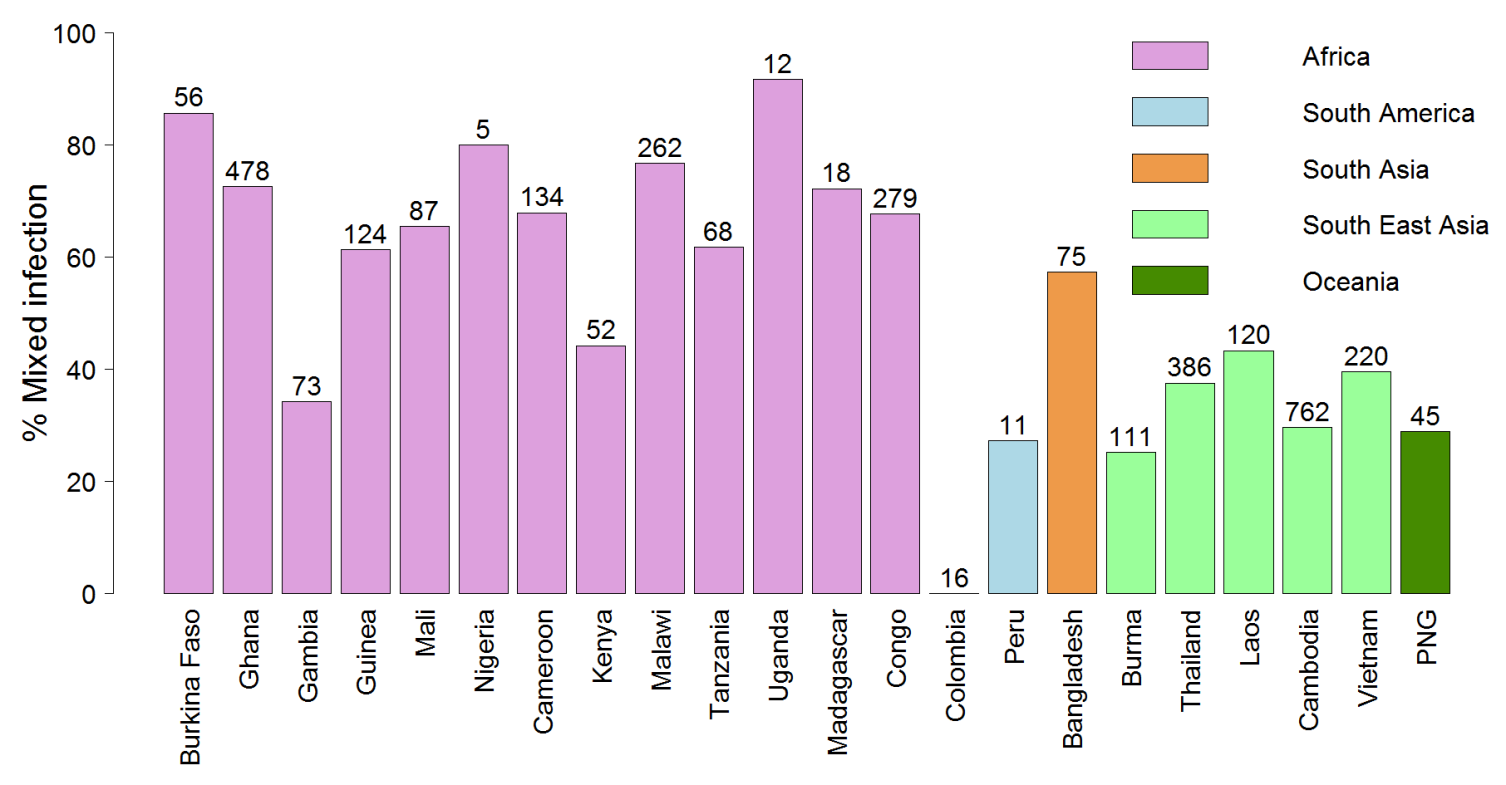
Lower estimated rate of mixed strain infections in samples from SEA. Mixed strain infection defined as samples with >10% SNP calls as heterozygous. This cut-off was determined from the distribution of heterozygous SNPs across the samples ( Supplementary Figure 3). The numbers of samples from each country are shown at the top of bar plots. PNG - Papua New Guinea.

**Supplementary Figure 1.**
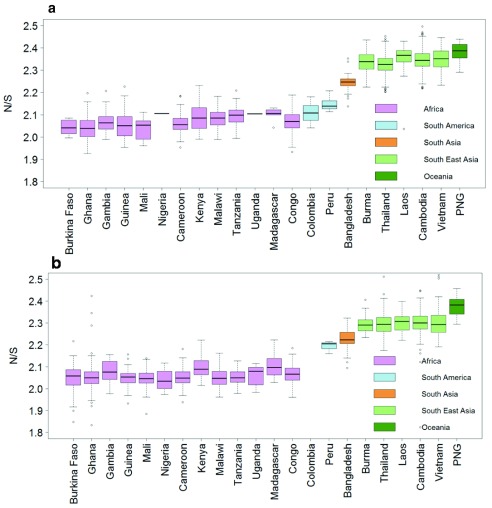
Higher ratio of non-synonymous to synonymous polymorphism in *P. falciparum* samples from SEA when single strain and mixed strain samples are analysed separately.. **A**) Box-plot showing N/S for 1670 single strain samples. There was only one single strain sample each from Nigeria and Uganda. **B**) Box-plot showing N/S for 1724 mixed strain samples. There were no mixed strain samples from Colombia. Mixed strain samples defined as samples with >10% SNP calls as heterozygous. This cut-off was determined from the distribution of heterozygous SNPs across the samples ( Supplementary Figure 3).

Blood samples may contain more than one haploid parasite clone due to mixed infections by multiple genotypes. The rate of mixed strain infection is generally lower in areas of low-transmission such as SEA ^20^. The lower efficiency of negative selection in removing potentially deleterious mutations at conserved positions in SEA could result from lower competition between parasite clones in the hosts. Indeed, the estimated rate of mixed strain infections, detected by a high proportion of heterozygous calls in the sequencing data, was much lower in South-East Asia compared to Africa ( Figure 6). We also confirmed that N/S is SEA samples was higher than samples from Africa even when separately analysing predicted single strain and mixed strain samples ( Supplementary Figure 1).

## Discussion

**Supplementary Figure 2.**
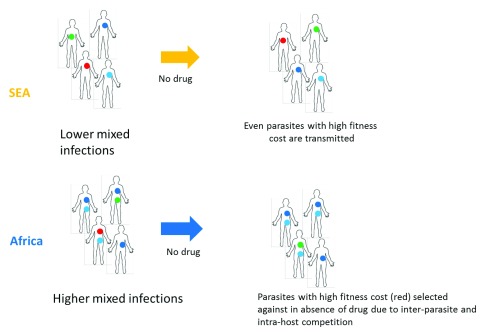
A model for the higher propensity of SEA populations to acquire drug resistance. Lower mixed infections in SEA may allow even less-fit parasites to be transmitted to next set of hosts due to reduced level of intra-host inter-parasite competition. Thus, fitness-reducing mutations including drug-resistance mutations might have a higher chance of spreading in SEA. In contrast, the higher mixed infection rate in Africa may drive more intense inter-clone competition within the host, thereby reducing the probability of transmission of the less-fit clones, including potentially drug-resistant clones. The public domain image of the outline of the human body was obtained from https://en.wikipedia.org/wiki/File:Outline-body.png.

Here we find a higher N/S ratio in strains from SEA compared to Africa. We also find that non-synonymous mutations have a higher likelihood to occur at conserved sites in SEA strains compared to African strains. In addition, we confirm a lower rate of mixed strain infection in SEA compared to Africa in the MalariaGEN dataset, the largest whole-genome dataset on *P. falciparum* till date. Based on these three observations, we propose a model for the higher propensity of SEA populations to acquire drug resistance ( Supplementary Figure 2). Lower mixed strain infections in SEA may allow even less-fit parasites to be transmitted to the next set of hosts due to reduced level of intra-host competition. In contrast, the higher mixed strain infection rate in Africa may drive more intense intra-host competetion, and may therefore reduce the probability of transmission of less-fit parasites. Thus, fitness-reducing mutations including drug-resistance mutations might have a higher chance of spreading in SEA compared to Africa in patients not taking drugs. Since Africa has higher rate asymptomatic infections as well as untreated patients, this would also result in higher competition between drug resistant and drug sensitive clones in the absence of drug, further decreasing the spread of drug resistance mutations with a fitness cost.

This model is consistent with a number of previous studies. Our observation of higher likelihood of fixation of potentially deleterious mutations in *P. falciparum* strains from SEA compared to African strains is consistent with the previous observation of higher rate of potentially deleterious copy number variations in *P. falciparum* from SEA compared to Africa ^21^. These observations suggest relaxed negative selection on *P. falciparum* from SEA compared to Africa and that SEA strains would have lower fitness than African strains. It would be fascinating to test this hypothesis experimentally e.g. by measuring the competitive asexual growth rate (an important component of fitness) of SEA and African *P. falciparum* strains.

Mixed strain infection by *P. falciparum* has recently been demonstrated to lead to within-host competition in patients ^22^, the possible mechanisms of which might include strain-transcending immunity, resource competition (e.g. RBCs) or direct interference between strains ^23– 26^. While within-host competition seems to be the major explanation for lower N/S in African strains, mixed strain infection would also lead to higher rate of recombination between gametes of different genotypes and efficient removal of deleterious mutations in Africa. In any case, a higher rate of mixed strain infection is expected to increase the strength of purifying selection.

What are the implications of our model for the current wave of artemisinin resistance? The much larger population size of *P. falciparum* in Africa ^21^, as also evidenced by the high rate of mixed strain infection ( Figure 6) should make it easier for resistance mutations to appear. Indeed, artemisinin resistance mutations in *kelch13*gene were observed in samples from Africa, including the most common artemisinin resistance mutation C580Y ^18^. The C580Y mutation is capable of generating artemisinin resistance *in vitro* in the NF54 parasite strain considered to be of African origin ^27^. This raises an important question as to why artemisinin resistance is not spreading in Africa. Since artemisinin resistance is likely to incur a fitness cost in the drug-free environment ^28– 30^, we propose that strains with these mutations are continuously arising in Africa but get competitively removed by the fitter drug-sensitive strains ^30^ in hosts not taking artemisinin. This effect might be pronounced by the greater proportion of asymptomatic and untreated patients in Africa. However once a strain acquires compensatory mutations that may reduce the fitness cost of the original mutation, it may be able to spread in a more competitive environment in Africa. While compensatory mutations can occur anywhere in the genome and may even spread in South-East Asia, these could be unlinked by recombination in areas with high transmission rate such as Africa ^31^. Thus, compensatory mutations in the same gene might be more likely to spread in high-transmission areas. Indeed, drug-resistance genes often acquire multiple mutations before spreading to Africa, *e.g.* pyrimethamine resistance gene *dhfr* acquired at least three different mutations in South-East Asia before it spread to Africa ^9^. All chloroquine-resistant strains have the K76T mutation in CRT (chloroquine-resistance transporter) but are accompanied by a number of mutations in the same gene ^32^. While at present *kelch13* does not appear to have multiple mutations ^33^, it would be critical to monitor the acquisition of additional mutations in the *kelch13* which might compensate the fitness cost of *kelch13* resistant mutations in the drug-free environment. Resistance to chloroquine and sulphadoxine-pyrimethamine spread from SEA to India to Africa ^3^. Interestingly we observed a higher mixed strain infection rate in Bangladesh than in neighboring SEA. The Indian subcontinent has areas with widely variable transmission rates ^34^. This might allow drug-resistant *P. falciparum* evolved in low transmission areas in SEA to gradually adapt to higher transmission areas in the Indian subcontinent, which could then spread to the high transmission areas in Africa. Therefore, it would be critical to track the spread of artemisinin resistance in the Indian subcontinent.

It is important to note that higher N/S in SEA populations does not necessarily imply higher mutation rate. Brown *et al.* previously found similar substitution rates in samples from Africa and SEA ^35^. Mutation rate as measured by long-term *in vitro* culture was not higher in strains from SEA origin, either in the presence or absence of drug ^36, 37^. Thus mutation rate in SEA population appears to be similar to that of African population, but a higher fraction of mutations are observed at conserved non-synonymous positions in SEA. The MalariGEN study from where we obtained the dataset reported much higher density (per sample) of both synonymous and non-synonymous polymorphisms in Africa compared to SEA ^18^. It is also important to note that higher density of SNP/sample does not imply higher substitution rate in Africa, rather it reflects the higher rate of mixed strain infection in Africa, *i.e.* more SNPs are identified in samples from Africa because of the higher number of different parasite clones per samples ( Figure 6). The authors of the MalariaGEN also wrote that at the gene level “we found virtually identical distributions of the ratio of non-synonymous to synonymous mutations (N/S ratio) in the two regions” ^18^, however, no statistical test was performed by the authors. Furthermore, no comparison of N/S at the sample level was performed in the MalariaGEN study. Resistance to chloroquine and sulfadoxine-pyrimethamine appeared independently in SEA and South America ^38^. While there were few samples from South-America in the MalariaGEN dataset ( Figure 6), we find that these samples also display lower mixed infection rate ( Figure 6), and N/S ratio in between the African and SEA samples ( Figure 1). Further analyses of a larger number of samples from South America could shed light on whether the mechanism we propose for a higher rate of resistance emergence in SEA might be applicable to South-America.

In summary, we propose that the lower transmission rates in SEA lead to a lower rate of mixed strain infection, which leads to reduced strength of natural selection. This, in turn, allows a higher rate of fixation of potentially deleterious mutations including drug resistance mutation. However, other factors such as drug usage, the level of immunity, and social factors ^3, 5, 39^, could also contribute towards the faster development of resistance in SEA. Given the basic difference in the transmission rate between SEA and Africa, which is not easy to control, we should expect that SEA would remain a source of drug resistance malaria in the future.

## Methods

**Supplementary Figure 3.**
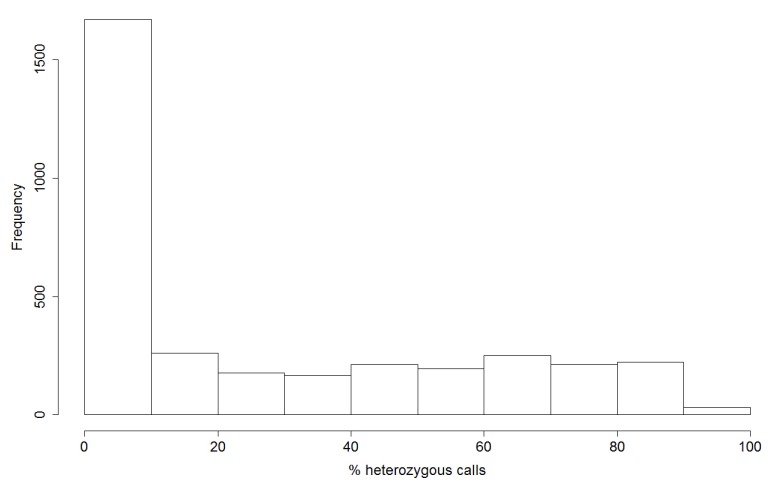
The frequency distribution of percentage heterozygous calls in samples. About half of the 3,394 samples showed percentage heterozygous calls less than ten percent and were defined as single strain samples. Rest of the samples showed roughly uniform distribution of percentage heterozygous calls and were defined as mixed strain samples.

The SNP data of *P. falciparum* was obtained from the MalariaGen community webpage ( https://www.malariagen.net/data/p-falciparum-community-project-jan-2016-data-release) ^18^. The SNP data consist of filtered and high quality 939,687 exonic SNPs with 631,715 non-synonymous and 307,972 synonymous SNPs. The data comprised 3,394 samples from 22 countries, with roughly equal number of samples from South-East Asia (1,600 samples) and Africa (1,647 samples). The N/S ratio for each sample was obtained by dividing the number of non-synonymous SNPs by the number of synonymous SNPs in that sample. Proteome sequences of *P. falciparum*, *P. berghei, P. chabaudi, P. cynomolgi, P. knowlesi, P. reichenowi, P. vivax, P. yoelii* were downloaded from PlasmoDB database and proteome sequences of *S. cerevisiae*, *D. melanogaster*, *C. elegans* and *H. sapiens* were downloaded from European Bioinformatics Institute (EBI) database. Orthologous sequences were identified using best bidirectional hit algorithm ^40^ and aligned using ClustalO ^41^. The conservation score for *P. falciparum* proteins was calculated as the percentage of positions identical across all orthologous proteins from *Plasmodium* species. The N/S ratio for each gene in South-East Asia and Africa was calculated by dividing the number of unique non-synonymous SNPs by the number of unique synonymous SNPs across samples from the two geographical areas. There were 136 genes with zero synonymous SNPs in SEA and thus were excluded from the analyses. The Pearson correlation between N/S for each gene and the conservation score was calculated in R. All figures were created in R version 3.2.3. Mixed infection samples were defined as samples with >10% SNP calls as heterozygous. This cut-off was determined from the distribution of heterozygous SNPs across the samples ( Supplementary Figure 3). It is important to note that this method is not likely to accurately classify each sample into a polyclonal (mixed infection) or a monoclonal sample, but the overall trend of higher rate of mixed infection in African samples compared to SEA samples is likely to be robust.

## Data availability

The data referenced by this article are under copyright with the following copyright statement: Copyright: © 2016 Singh GP and Sharma A

This publication uses data from the MalariaGEN *Plasmodium falciparum* Community Project as described in Genomic epidemiology of artemisinin resistant malaria, eLife, 2016 (DOI: http://dx.doi.org/10.7554/eLife.08714). This data is also available from the MalariaGEN website ( https://www.malariagen.net/data/p-falciparum-community-project-jan-2016-data-release).

## Figures and Tables

**Supplementary Table 1.** List of functionally annotated genes with more than 3-fold higher N/S in SEA than Africa and N/S in SEA more than 4.

Geneld	Description	N/S SEA	N/S Africa	Fold Higher in SEA
PF3D7_0709000	chloroquine resistance transporter (CRT)	10.3	1.3	8.1
PF3D7_0302700	CDGSH iron-sulfur domain-containing protein	10.0	1.4	7.2
PF3D7_0816200	vacuolar protein-sorting protein VPS2	6.0	0.9	6.4
PF3D7_0410400	exosome complex component RRP4 (RRP4)	7.0	1.2	5.9
PF3D7_0922900	beta-ketoacyl-acyl carrier protein reductase (FabG)	11.0	2.0	5.5
PF3D7_0314100.1	vesicle transport v-SNARE protein	6.0	1.1	5.5
PF3D7_1124800	nuclear preribosomal assembly protein	8.0	1.5	5.4
PF3D7_0936000	ring-exported protein 2+(REX2)	13.0	2.6	5.1
PF3D7_0720000	3'-5'+exoribonuclease Csl4 homolog	6.0	1.2	5.1
PF3D7_0110900	adenylate kinase	8.0	1.7	4.8
PF3D7_1313700	septum formation protein MAF homologue	10.0	2.1	4.7
PF3D7_0304600	circumsporozoite (CS) protein (CSP)	12.0	2.7	4.5
PF3D7_0515100	rhomboid protease ROM9 (ROM9)	9.0	2.0	4.4
PF3D7_0109400	tubulin-specific chaperone a	7.0	1.6	4.3
PF3D7_0522700	iron-sulfur assembly protein (SufA)	8.0	1.9	4.1
PF3D7_0702300	sporozoite threonine and asparagine-rich protein (STARP)	15.5	3.9	4.0
PF3D7_1435000	cytochrome c+oxidase assembly protein	9.0	2.3	3.9
PF3D7_0109300	fatty acid elongation protein, GNS1/SUR4 family	4.5	1.2	3.9
PF3D7_1410100	alpha/beta hydrolase	5.3	1.4	3.9
PF3D7_0319600	elongation factor 1+(EF-1)	7.0	1.8	3.9
PF3D7_1143200	DnaJ protein	14.0	3.6	3.8
PF3D7_1028300	nucleolar preribosomal assembly protein	13.0	3.5	3.8
PF3D7_1358300	rhomboid protease ROM7 (ROM7)	5.5	1.5	3.7
PF3D7_1343700	kelch protein	4.2	1.1	3.7
PF3D7_0416100	glutamyl-tRNA(Gln) amidotransferase subunit A	8.0	2.2	3.6
PF3D7_0423400	asparagine-rich protein (AARP)	9.0	2.5	3.5
PF3D7_1210900	GPI mannosyltransferase I+(PIG-M)	5.0	1.4	3.5
PF3D7_1015800	ribonucleotide reductase small subunit	5.0	1.5	3.3
PF3D7_1217600	anaphase promoting complex subunit 10	6.0	1.9	3.2
PF3D7_1143300	DNA-directed RNA polymerase I	4.3	1.4	3.1
PF3D7_1405200	sybindin-like protein	5.0	1.6	3.1
PF3D7_1023400	HORMA domain protein	8.0	2.6	3.1
PF3D7_1434000	CCR4-associated factor 16 (CAF16)	6.5	2.1	3.1
