# Supplementary material for: South-East Asian strains of Plasmodium falciparum display higher ratio of non-synonymous to synonymous polymorphisms compared to African strains
Source: F1000Res. 2016 Oct 21;5:1964. Originally published 2016 Aug 12. [Version 2] doi: 10.12688/f1000research.9372.2 (PMC5089136; doi:10.12688/f1000research.9372.2)
Supplement: Peer review history

# Peer review: Referee response for version 2

**DOI:** 10.5256/f1000research.10627.r17143

**Published:** 2016-10-27

**Authors:** Daniel N. Hupalo

**Affiliations:** 1 Center for Genomics and Systems Biology, Department of Biology, New York University, New York, NY, USA

This revised article "South-East Asian strains of *Plasmodium falciparum* display higher ratio of non-synonymous to synonymous polymorphisms compared to African strains" address several of the different criticisms listed by the reviewer in the recent submitted peer-review. In particular, the authors have revised their discussion section to better highlight the contrast between their conclusions and the conclusions made by a similar MalariaGen analysis. The authors have also addressed errors highlighted by the reviewer in both figure legends and discussion concerning the effects of selection. Together, these changes create a stronger article worth being indexed.

I have read this submission. I believe that I have an appropriate level of expertise to confirm that it is of an acceptable scientific standard.

# Peer review: Referee response for version 1

**DOI:** 10.5256/f1000research.10094.r16468

**Published:** 2016-09-21

**Authors:** Tim J. Anderson

**Affiliations:** 1 Department of Genetics, Texas Biomedical Research Institute, San Antonio, TX, USA

- ***Title and Abstract***

The title is appropriate.

 The abstract makes some jumps in logic:  e.g *“*Furthermore, South-East Asian strains showed a higher proportion of non-synonymous polymorphism at conserved positions, suggesting reduced negative selection.” This pattern can equally well be explained by stronger positive selection in SEA than in Africa.

  

- ***Article content***

 

Major points

Results differ from the prior analysis of this dataset. The article comes to different conclusions from those reported by the MalariaGEN consortium in eLife (ref 18) who write: *"*Accordingly, we found virtually identical distributions of the ratio of non-synonymous to synonymous mutations (N/S ratio) in the two regions ( Figure 3c)” page 6 bottom: MalariaGEN *Plasmodium falciparum* Community Project. eLife 2016;5:e08714. DOI: 10.7554/eLife.08714). It is unclear to me why this F1000 submission and the published eLife paper reach very different conclusions using the same dataset? I suggest that the authors should cite the conclusions of the eLife paper and explain why their analysis comes to a very different conclusion. This point needs urgently needs satisfactory resolution as this is the central conclusion of the paper.

Methodology/analysis biases. Reduced ability to score non-synonymous variants in African populations could generate the N/S ratios observed. Such a bias could be generated because infections are more complex in Africa. Non-synonymous mutations tend to be at lower frequency than synonymous mutations. In complex mixed African infections low frequency non-synonymous mutations may be filtered out of the dataset (because very few reads show the mutation) but scored in SE Asian samples, where infections are simpler (so multiple reads show non-synonymous mutations). To eliminate this potential methodological bias, the authors should filter their data to minimize mixed infections prior to analysis. This could be done by setting a threshold for numbers of mixed base calls. Again, it is important to understand why the conclusions reached by this analysis differ from the published eLife paper.

 As pointed out in the eLife paper there is a dramatic difference in the allele frequency spectrum in the two locations. The excess of rare variants in Africa could also potentially alter the N/S ratios.

Population genetics explanation. Assuming the central result is correct, a simpler model explains these results: The effective population size (Ne) of parasite populations is lower in SEA than in Africa. Therefore purifying selection is weaker in SEA than in Africa resulting in less efficient removal of deleterious mutations. Discussion of this simple alternative explanation would improve the paper. Also Cheeseman *et al, *MBE 2016 (Mol Biol Evol. 2016 Mar;33(3):603-20. doi: 10.1093/molbev/msv282.), who make this argument to explain patterns of global copy number polymorphism in *Plasmodium falciparum*.

Statistics used: The paper counts numbers of non-synonymous and synonymous mutations and estimates the ratio (N/S). This is an unusual way to present such data – it would be more informative to show dN/dS – (Nonsynonymous changes per non-synonymous site/synonymous variants per synonymous site). This statistic is more useful because the expected ratio under neutrality is 1. This should not change the results but will be more easily interpretable with reference to a neutral model.

 Related questions: How is N/S ratio determined for genes in which there are NO synonymous mutations? Details of the methods for calculating this ratio, and how they differ from those used in the eLife paper should be provided.

More support needed for intrahost competition explanation: The authors argue that the observed pattern may result from more within infection competition in Africa relative to Asia. If competition is invoked to explain the results, then it would be useful to examine the categories of genes contributing to the excess of NS variations. This argument would imply that this effect should be seen in subsets of genes involved in within host competition. On the other hand, if there is no particular enrichment of particular gene classes, then the simple population genetics explanation seems more likely.

  

- ***Conclusions***

 

Lack of balance. The authors argue that drug resistance may arise in SEA rather than Africa because intrahost competition prevents emergence of resistance mutations associated with fitness costs. I agree in part – this may certainly contribute. However, greater selection for resistance to drugs in SE Asia is likely to be a critical factor. Most infections in SEA are symptomatic and infected individuals seek treatment. However, most infections in Africa are asymptomatic, so people to no seek treatment. Discussion of the difference in selection strength between continents would add balance to this paper.

Other issues. I was very surprised to see this analysis of MalariaGEN data without involvement from malariaGEN authors. My understanding is that access to these data requires a "Fort Lauderdate" type agreement with MalariaGEN (https://www.malariagen.net/data/terms-use/pf3k-terms-use). The authors may have already discussed this with relevant people at MalariaGEN - I would suggest that the authors directly contact Dominic Kwiatkowski, if they have not already done so.

I have read this submission. I believe that I have an appropriate level of expertise to state that I do not consider it to be of an acceptable scientific standard, for reasons outlined above.

# Response

**Published:** 2016-10-17

**Authors:** Gajinder Singh

**Affiliations:** International Centre for Genetic Engineering and Biotechnology, New Delhi, India

We are grateful for your valuable comments, which we believe have significantly improved our manuscript.

*The abstract makes some jumps in logic:  e.g “Furthermore, South-East Asian strains showed a higher proportion of non-synonymous polymorphism at conserved positions, suggesting reduced negative selection.” This pattern can equally well be explained by stronger positive selection in SEA than in Africa.*

**Author response: **Positive selection can indeed increase (though not in all cases) N/S for the gene under selection, as has been observed for the *kelch13* gene in SEA. However, the pattern of higher N/S in strains in SEA which can be observed at genome-wide level is much more consistent with relaxed negative selection in SEA, rather than higher positive selection in SEA, which typically acts at a few sites. Furthermore, reduced constraints at conserved sites at the genome-wide level supports relaxed negative selection rather than positive selection. We thus believe that our use of word “suggesting” is appropriate in the statement.

- ***Article content***

 

*Major points*

**

*Results differ from the prior analysis of this dataset. The article comes to different conclusions from those reported by the MalariaGEN consortium in eLife (ref 18) who write: "Accordingly, we found virtually identical distributions of the ratio of non-synonymous to synonymous mutations (N/S ratio) in the two regions (* *Figure 3c* *)” page 6 bottom: MalariaGEN Plasmodium falciparum Community Project. eLife 2016;5:e08714. DOI: 10.7554/eLife.08714). It is unclear to me why this F1000 submission and the published eLife paper reach very different conclusions using the same dataset? I suggest that the authors should cite the conclusions of the eLife paper and explain why their analysis comes to a very different conclusion. This point needs urgently needs satisfactory resolution as this is the central conclusion of the paper.*

**Author response: **The authors in the MalariaGEN study did not perform any statistical test to support their statement that N/S in SEA and Africa is similar at the gene level. While the difference is subtle, it is highly statistically significant, as we report in our manuscript. Furthermore, the authors in MalariaGEN study did not analyse differences in N/S at the sample level, where we clearly see the differences between SEA and African strains (Figure 1). We have now added these statements to the Discussion section of the revised manuscript.

*Methodology/analysis biases. Reduced ability to score non-synonymous variants in African populations could generate the N/S ratios observed. Such a bias could be generated because infections are more complex in Africa. Non-synonymous mutations tend to be at lower frequency than synonymous mutations. In complex mixed African infections low frequency non-synonymous mutations may be filtered out of the dataset (because very few reads show the mutation) but scored in SE Asian samples, where infections are simpler (so multiple reads show non-synonymous mutations). To eliminate this potential methodological bias, the authors should filter their data to minimize mixed infections prior to analysis. This could be done by setting a threshold for numbers of mixed base calls. Again, it is important to understand why the conclusions reached by this analysis differ from the published eLife paper.*

**

**Author response: **Separately analysing predicted monoclonal and polyclonal samples did not change conclusions (Supplementary Figure 1 in the revised manuscript). This is not surprising given that we observed almost no overlap in the distributions of N/S in SEA and African samples (Figure 1), as we wrote in the manuscript. We would like to emphasize that authors in MalariaGEN study did not perform N/S analyses at the sample level.

*As pointed out in the eLife paper there is a dramatic difference in the allele frequency spectrum in the two locations. The excess of rare variants in Africa could also potentially alter the N/S ratios.*

**Author response: **As proposed by the reviewer in the previous comment, the excess of rare variants in Africa might increase N/S in Africa, thus the higher N/S in SEA could not be explained by excess rare variants in Africa. 

*Population genetics explanation. Assuming the central result is correct, a simpler model explains these results: The effective population size (Ne) of parasite populations is lower in SEA than in Africa. Therefore purifying selection is weaker in SEA than in Africa resulting in less efficient removal of deleterious mutations. Discussion of this simple alternative explanation would improve the paper. Also Cheeseman et al, MBE 2016 (MolBiolEvol. 2016 Mar;33(3):603-20. doi: 10.1093/molbev/msv282.), who make this argument to explain patterns of global copy number polymorphism in Plasmodium falciparum.*

**

**Author response:** Indeed relaxed negative selection in SEA due to lower effective population (reflected in lower polyclonal infections) is exactly what we propose in our manuscript for our observation of higher N/S in SEA. Lower Ne (effective population size) is bound to be associated with lower polyclonal infection rate, which has been shown to lead to within-host competition in *P. falciparum* ^1^. Thus we do not believe that the two explanations are alternative independent explanations, one of which needs to be discounted. Rather the observation made by Cheeseman *et al,* and in our manuscript are convergent which further supports reduced negative selection in *P. falciparum* in SEA and we have added these statements to the Discussion section of the revised manuscript.

*Statistics used: The paper counts numbers of non-synonymous and synonymous mutations and estimates the ratio (N/S). This is an unusual way to present such data – it would be more informative to show dN/dS – (Nonsynonymous changes per non-synonymous site/synonymous variants per synonymous site). This statistic is more useful because the expected ratio under neutrality is 1. This should not change the results but will be more easily interpretable with reference to a neutral model.*

**

**Author response: **As pointed out by the reviewer dN/dS comparison would not change the results. *P. falciparum* is unusual in having very high N/S compared to other organisms ^2^, thus the usual expectation of dN/dS of 1 for neutral evolution is not applicable for *P. falciparum*.

*Related questions: How is N/S ratio determined for genes in which there are NO synonymous mutations? Details of the methods for calculating this ratio, and how they differ from those used in the eLife paper should be provided.*

**Author response: **There were 136 genes with zero synonymous SNPs in SEA (none in Africa) and thus were excluded from the analyses. We have added this statement in the Method section of the revised manuscript.

*More support needed for intrahost competition explanation: The authors argue that the observed pattern may result from more within infection competition in Africa relative to Asia. If competition is invoked to explain the results, then it would be useful to examine the categories of genes contributing to the excess of NS variations. This argument would imply that this effect should be seen in subsets of genes involved in within host competition. On the other hand, if there is no particular enrichment of particular gene classes, then the simple population genetics explanation seems more likely.*

**Author response: **There is no dataset of genes known to be involved in within-host competition in *P. falciparum*. We do note however that many housekeeping genes show higher N/S in SEA than Africa (Supplementary Table 1). As discussed in response to previous comments by the reviewer, the lower Ne, lower rate of polyclonal infections and within-host completion are not mutually exclusive explanations, one of which needs to be discounted.

  

- ***Conclusions***

 

*Lack of balance. The authors argue that drug resistance may arise in SEA rather than Africa because intrahost competition prevents emergence of resistance mutations associated with fitness costs. I agree in part – this may certainly contribute. However, greater selection for resistance to drugs in SE Asia is likely to be a critical factor. Most infections in SEA are symptomatic and infected individuals seek treatment. However, most infections in Africa are asymptomatic, so people to no seek treatment. Discussion of the difference in selection strength between continents would add balance to this paper.*

**

**Author response: **The higher rate asymptomatic infections as well as untreated patients in Africa would mean higher competition between drug resistant and drug sensitive clones in the absence of drug, further decreasing the spread of drug resistance mutations with a fitness cost. Thus higher immunity, lower treatment rates, and higher polyclonal infections are likely to work synergistically. We have added these statements to the Discussion section in the revised manuscript.   

**

*Other issues. I was very surprised to see this analysis of MalariaGEN data without involvement from malariaGEN authors. My understanding is that access to these data requires a "Fort Lauderdate" type agreement with MalariaGEN (https://www.malariagen.net/data/terms-use/pf3k-terms-use). The authors may have already discussed this with relevant people at MalariaGEN - I would suggest that the authors directly contact Dominic Kwiatkowski, if they have not already done so.*

**Author response: **We have taken permission from the authors of the MalariaGEN study, and their contribution has been appropriately acknowledged in our publication.

 We would again like to thank you for your critical comments on our manuscript, and would be happy to address any further concerns that you may have in the revised version of the manuscript.

 Reference List

     1.    Bushman, M. *et al.* Within-host competition and drug resistance in the human malaria parasite Plasmodium falciparum. *Proc. Biol. Sci.* **283**, 20153038 (2016).

     2.    Chang, H. H. *et al.* Malaria life cycle intensifies both natural selection and random genetic drift. *Proc. Natl. Acad. Sci. U. S. A.* **110**, 20129-20134 (2013).

# Peer review: Referee response for version 1

**DOI:** 10.5256/f1000research.10094.r15909

**Published:** 2016-09-20

**Authors:** Georges Snounou

**Affiliations:** 1 Sorbonne Universités, University Pierre and Marie Curie Univ Paris 06, National Institute of Health and Medical Research, Centre of Immunology and Infectious Diseases, French National Center for Scientific Research, Paris, France

In this article the authors conduct a detailed analysis that focused on the frequency of non-synonymous mutations across an extensive genome set derived by the MalariaGEN consortium from an extensive set of *P. falciparum* isolates collected mainly in Africa and SE Asia. The analyses extend the work presented recently (DOI: 10.7554/eLife.08714) where a trend was noted for a higher level of non-synonymous mutations in the SE Asian parasites a compared to that in the African parasites. Specifically, the authors have presented the analysis for the parasites from individual countries and focused on SNPs that occur at residues that are highly conserved in orthologues within a variety of *Plasmodium* species and across eukaryotes in general. The authors have thus provided convincing evidence for statistically significant albeit relatively subtle differences between the parasites from SE Asia and Africa (it is unclear to me why parasites from Peru and Colombia were included in the analysis, all the more as their numbers were relatively low).

 Whereas, the quality of the data, the analyses and the results are not into question, I have some misgivings and indeed frustrations as to the conclusions put forward in an attempt to account for the differences observed. The scenario inspired by the observations that the authors propose as an explanation for the propensity of antimalarial drug resistance to emerge and spread in *P. falciparum* from SE Asia first before doing so in Africa is plausible and could be supported by the data presented. However, this scenario is based on a set of assumptions whose limitations are not discussed, without any justification as to why this particular scenario is the most likely.

  

1. What is the nature of the intra-host competition between the genotypes of *P. falciparum* that circulate in humans, and is there any evidence that this actually alters transmissibility? Immunity is the most obvious selective intra-host factor. To which extend differences in the levels of immunity among the patients that contributed the *P. falciparum* isolates analysed here alters the multiplicity of the infection or indeed the likelihood of genetic crosses?
2. Is there any evidence that parasite with the higher levels of non-synonymous mutations across the genome are biologically less fit? There is evidence that this is the case for drug resistant parasites *in vitro*, that for parasites growing *in vivo* is less straightforward and varies for different drugs.
3. To what extend are the isolates collected from a limited number of febrile patients reflect the overall parasite population across a whole country. Could a bias be introduced simply because of differences in the degree of acquired immunity on admission, the time between the onset of the infection and the time treatment was sought, or the level of admission parasitaemia?
4. Exposure to drug is clearly a strong selective constraint for those genes implicated in overcoming the effects of the drug. What can be the nature of the selective constrains that maintain the higher levels of non-synonymous mutations across numerous genes spread throughout the genome? Clearly not all can be considered to provide some speculative compensatory effect to a potential reduction in fitness following drug selection.
5. It should be pointed out that in the 1950’s resistance to chloroquine first appeared not only in SE Asia but also in Colombia. Similarly resistance to pyrimethamine did not first appear in SE Asia, but in all areas where the use of this drug became widespread and systematic (this was the case in both SE Asia and African countries in the 1950’s). The “unanswered” question as to the reason why drug resistance has repeatedly emerged in SE Asia, might have more to do with drug usage than with some special property of the parasites.

 

 Ultimately, I would suggest that the authors should present the limitations of their conclusions as well as alternative scenarios to account for their observations (and rank or discount them if they can). This will surely be welcome by readers. Advances in knowledge are seeded by diversity in speculation (conservative or less so) and genome wide analyses are a rich source for this.

I have read this submission. I believe that I have an appropriate level of expertise to confirm that it is of an acceptable scientific standard, however I have significant reservations, as outlined above.

## References

[1] MalariaGEN Plasmodium falciparum Community Project: Genomic epidemiology of artemisinin resistant malaria. *Elife*.2016;5: 10.7554/eLife.08714 10.7554/eLife.08714 26943619PMC4786412

# Response

**Published:** 2016-10-17

**Authors:** Gajinder Singh

**Affiliations:** International Centre for Genetic Engineering and Biotechnology, New Delhi, India

We are grateful for your valuable comments, which we believe have significantly improved our manuscript.

*In this article the authors conduct a detailed analysis that focused on the frequency of non-synonymous mutations across an extensive genome set derived by the MalariaGEN consortium from an extensive set of P. falciparum isolates collected mainly in Africa and SE Asia. The analyses extend the work presented recently (DOI: 10.7554/eLife.08714) where a trend was noted for a higher level of non-synonymous mutations in the SE Asian parasites a compared to that in the African parasites. Specifically, the authors have presented the analysis for the parasites from individual countries and focused on SNPs that occur at residues that are highly conserved in orthologues within a variety of Plasmodium species and across eukaryotes in general. The authors have thus provided convincing evidence for statistically significant albeit relatively subtle differences between the parasites from SE Asia and Africa (it is unclear to me why parasites from Peru and Colombia were included in the analysis, all the more as their numbers were relatively low).*

**Author response: **We would like to clarify that MalariaGEN authors did not write or suggest that N/S ratio of *P. falciparum *strains from SEA and Africa are different. We have discussed the difference between the work reported in MalariaGEN study and our manuscript in the Discussion section of the revised manuscript. We added data from Peru and Columbia in Figures 1, Figure 5 and Figure 6 only for completeness, but we have refrained from making any firm conclusions based on these samples. We have nevertheless commented upon the observations from South-American samples in the Discussion section in response to your comment.

*Whereas, the quality of the data, the analyses and the results are not into question, I have some misgivings and indeed frustrations as to the conclusions put forward in an attempt to account for the differences observed. The scenario inspired by the observations that the authors propose as an explanation for the propensity of antimalarial drug resistance to emerge and spread in P. falciparum from SE Asia first before doing so in Africa is plausible and could be supported by the data presented. However, this scenario is based on a set of assumptions whose limitations are not discussed, without any justification as to why this particular scenario is the most likely.*

- *What is the nature of the intra-host competition between the genotypes of P. falciparum that circulate in humans, and is there any evidence that this actually alters transmissibility? Immunity is the most obvious selective intra-host factor. To which extend differences in the levels of immunity among the patients that contributed the P. falciparum isolates analysed here alters the multiplicity of the infection or indeed the likelihood of genetic crosses?*

**Author response: **We thank the reviewer for pointing this out. There is indeed evidence of intra-host competition between genotypes of *P. falciparum *in humans that reduces parasite density ^1^. The association between parasite density and gametocyte density in humans has also been previously shown ^2^. The reasons for intra-host competition however remain unknown. Strain-transcending immunity, resource competition (e.g. RBCs), and direct interference between strains have been proposed as possible mechanisms responsible for within-host competition ^3-6^. It is also possible that lower recombination rates due to lower rate of mixed infections in SEA may reduce the removal of deleterious mutations. We have added these sentences to the Discussion section of the revised manuscript.

- *Is there any evidence that parasite with the higher levels of non-synonymous mutations across the genome are biologically less fit? There is evidence that this is the case for drug resistant parasites in vitro, that for parasites growing in vivo is less straightforward and varies for different drugs.*

**Author response: **There is currently no evidence that *P. falciparum *strains from SEA are biologically less fit that strains from Africa. Indeed that is the prediction of our model. It would be fascinating to test this hypothesis. We have added these sentences to the Discussion section of the revised manuscript.

- *To what extend are the isolates collected from a limited number of febrile patients reflect the overall parasite population across a whole country. Could a bias be introduced simply because of differences in the degree of acquired immunity on admission, the time between the onset of the infection and the time treatment was sought, or the level of admission parasitaemia?*

**Author response: **It is possible that samples collected may not be representative of the whole country; however the consistent results we obtain for across countries in Africa and SEA suggest that our observations of higher N/S is SEA compared to Africa is robust to random variations introduced due to sampling.

 It is possible that some biases in sampling may be introduced that could lead to differences in the observed rate of polyclonal infections between Africa and SEA. We separately analysed predicted monoclonal and polyclonal samples and find results similar to those obtained using all samples (Supplementary Figure 1 in the revised manuscript). We thus currently have no hypothesis of how biased sampling could actually lead to higher genome-wide N/S in SEA.

- *Exposure to drug is clearly a strong selective constraint for those genes implicated in overcoming the effects of the drug. What can be the nature of the selective constrains that maintain the higher levels of non-synonymous mutations across numerous genes spread throughout the genome? Clearly not all can be considered to provide some speculative compensatory effect to a potential reduction in fitness following drug selection. *

**Author response: **As further explained in the Discussion section, the higher genome scale N/S in SEA is consistent with relaxed negative selection. We hypothesise that relaxed negative selection could be due to low level of polyclonal infection and thus lower within-host competition in SEA. 

- *It should be pointed out that in the 1950’s resistance to chloroquine first appeared not only in SE Asia but also in Colombia. Similarly resistance to pyrimethamine did not first appear in SE Asia, but in all areas where the use of this drug became widespread and systematic (this was the case in both SE Asia and African countries in the 1950’s). The “unanswered” question as to the reason why drug resistance has repeatedly emerged in SE Asia, might have more to do with drug usage than with some special property of the parasites.*

**Author response: **Indeed resistance to chloroquine and sulphadoxine-pyrimethamine also appeared independently in South-America (but not in Africa ^7^). We have very few samples from South America (27 samples from 2 countries) to make robust conclusions. However it is interesting that samples from South America do show low rate of polyclonal infections and higher N/S compared to Africa (Figure 1 and Figure 6). We have added these sentences to the Discussion section of the revised manuscript.

*Ultimately, I would suggest that the authors should present the limitations of their conclusions as well as alternative scenarios to account for their observations (and rank or discount them if they can). This will surely be welcome by readers. Advances in knowledge are seeded by diversity in speculation (conservative or less so) and genome wide analyses are a rich source for this.*

**Author response: **We have significantly expanded the Discussion section which we hope addresses the reviewer’s concerns. We would be happy to address any further concerns that you may have in the revised version of the manuscript.

 Reference List

    1.   Bushman, M. *et al.* Within-host competition and drug resistance in the human malaria parasite Plasmodium falciparum. *Proc. Biol. Sci.* **283**, 20153038 (2016).

    2.   Ross, A., Killeen, G. & Smith, T. Relationships between host infectivity to mosquitoes and asexual parasite density in Plasmodium falciparum. *Am. J. Trop. Med. Hyg.* **75**, 32-37 (2006).

    3.   Metcalf, C. J. *et al.* Partitioning regulatory mechanisms of within-host malaria dynamics using the effective propagation number. *Science.* **%19;333**, 984-988 (2011).

    4.   Raberg, L. *et al.* The role of immune-mediated apparent competition in genetically diverse malaria infections. *Am. Nat.* **168**, 41-53 (2006).

    5.   Bruce, M. C. & Day, K. P. Cross-species regulation of malaria parasitaemia in the human host. *Curr. Opin. Microbiol.* **5**, 431-437 (2002).

    6.   Yap, G. S. & Stevenson, M. M. Blood transfusion alters the course and outcome of Plasmodium chabaudi AS infection in mice. *Infect. Immun.* **62**, 3761-3765 (1994).

    7.   Roper, C. *et al.* Intercontinental spread of pyrimethamine-resistant malaria. *Science.* **%20;305**, 1124 (2004).

# Peer review: Referee response for version 1

**DOI:** 10.5256/f1000research.10094.r15966

**Published:** 2016-09-08

**Authors:** Daniel N. Hupalo

**Affiliations:** 1 Center for Genomics and Systems Biology, Department of Biology, New York University, New York, NY, USA

This is a compelling manuscript from Gajinder Pal Singh and Amit Sharma, which conducts an analysis of the accrual of non-synonymous changes within *Plasmodium falciparum* ( *Pf*) genes across the breadth of the MalariaGen population genomic dataset. This work builds upon a trend observed in the initial MalariaGen publication(s) that South East Asian (SEA) populations exhibited a general trend of higher Non-synonymous to synonymous mutation ratio (N/S) compared to African populations. The authors replicate and expand this analysis and then compare this trend to a protein dataset comprised of Eukaryotic sequences to show that even among the most conserved sites SEA continues to accrue more NS polymorphisms than African isolates. Lastly the authors set out an argument for the mechanism behind this apparent relaxed negative selection within SEA *Pf*, attributing it to the lower rate of mixed genotype infections across SEA and the subsequent lack of competition within host. This would create the circumstances that would allow for multiple independent resistance mutations arising and fixing within the SEA population. Exploring general patterns of genome architecture within *P. falciparum* is vital to understanding the spread and prevention of resistance to anti-malarial drugs, and as a result this work is of interest to the malaria community. There are some clarifications required, and two significant areas of improvement for the paper:

 Major clarifications:

  

1. More detailed results/discussion sections, and deeper discussion of previous literature.While the authors cite the MalariaGen publication (citation 18) they should more explicitly describe the relationship between that publication and this analysis. Specifically, highlighting Figure 5 of citation 18 and its supplemental material, which shows a similar genome-wide analysis of N/S ratio between Africa/SEA. It would be good to directly acknowledge this article/figure as the first description of this African/SEA pattern. Furthermore, it would be constructive for the reader if the authors added a deeper justification for their extension of this work. 
2. Language used to describe the accrual of Nonsynonymous sites overstates the general trend of NS SNPs at conserved sites. Figure 4 shows that NS SNPs accrue at a higher rate in SEA across the set of conserved sites within *Plasmodium* and at the set of conserved sites across Eukaryotes. However, the language used to describe this figure in the legend states that “NS polymorphisms… in SEA are more likely to occur at conserved positions.” This statement implies that this is a general genome-wide trend of higher rate of fixing NS SNPs at sites with high conservation. Perhaps extending this legend to mention that NS SNPs are “are more likely to occur at conserved positions when compared to Africa” would avoid this implied generalization. Additionally, on page 3 the authors state “these mutations preferentially occur at the conserved sites”. This statement repeats the highlighted issue above, and adds the word “preferentially” which may create an unintentional teleological statement about the occurrences of NS mutations. NS SNPs occur at a higher rate within the pre-selected subset of conserved sites when compared to Africa, but the figures do not show a general genome-wide preference for NS mutations at conserved sites. 

Minor clarifications are as follows:

- “Kelch” is misspelled multiple times throughout the article.
- “Cambodia” is misspelled in multiple figures.
- “PNG” Abbreviation should be written out fully.

I have read this submission. I believe that I have an appropriate level of expertise to confirm that it is of an acceptable scientific standard, however I have significant reservations, as outlined above.

# Response

**Published:** 2016-10-17

**Authors:** Gajinder Singh

**Affiliations:** International Centre for Genetic Engineering and Biotechnology, New Delhi, India

*This is a compelling manuscript from Gajinder Pal Singh and Amit Sharma, which conducts an analysis of the accrual of non-synonymous changes within Plasmodium falciparum (Pf) genes across the breadth of the MalariaGen population genomic dataset. This work builds upon a trend observed in the initial MalariaGen publication(s) that South East Asian (SEA) populations exhibited a general trend of higher Non-synonymous to synonymous mutation ratio (N/S) compared to African populations. The authors replicate and expand this analysis and then compare this trend to a protein dataset comprised of Eukaryotic sequences to show that even among the most conserved sites SEA continues to accrue more NS polymorphisms than African isolates. Lastly the authors set out an argument for the mechanism behind this apparent relaxed negative selection within SEA Pf, attributing it to the lower rate of mixed genotype infections across SEA and the subsequent lack of competition within host. This would create the circumstances that would allow for multiple independent resistance mutations arising and fixing within the SEA population. Exploring general patterns of genome architecture within P. falciparum is vital to understanding the spread and prevention of resistance to anti-malarial drugs, and as a result this work is of interest to the malaria community. There are some clarifications required, and two significant areas of improvement for the paper:*

**Author response: **We thank you for your review and comments. We would like to clarify that higher N/S in *P. falciparum* strains from SEA compared to African strains has not been stated or suggested in the MalariaGen manuscript (Elife. 2016). In fact the authors of the MalariaGEN wrote that at the gene level “we found virtually identical distributions of the ratio of non-synonymous to synonymous mutations (N/S ratio) in the two regions”, however, no statistical test was performed by the authors. Furthermore, no comparison of N/S at the sample level was performed in the MalariaGEN study. We have added these statements to the Discussion section of the revised manuscript.

*Major clarifications:*


*  *

- *More detailed results/discussion sections, and deeper discussion of previous literature. While the authors cite the MalariaGen publication (citation 18) they should more explicitly describe the relationship between that publication and this analysis. Specifically, highlighting Figure 5 of citation 18 and its supplemental material, which shows a similar genome-wide analysis of N/S ratio between Africa/SEA. It would be good to directly acknowledge this article/figure as the first description of this African/SEA pattern. Furthermore, it would be constructive for the reader if the authors added a deeper justification for their extension of this work. *

**Author response: **The aim of Figure 5 of the MalariaGen manuscript (Elife. 2016) was to test whether neutral evolution could account for the pattern of mutations in *kelch13*. Figure 5a plots genic N/S vs. protein conservation in Africa and SEA. The Figure shows that *kelch13* gene is an outlier in the scatter-plot for SEA, but follows a general trend in Africa. Figure 5b plots the ratio of N/S ratios in SEA and Africa, for all genes (with ≥5 synonymous and ≥5 non-synonymous SNPs) and shows again that *kelch13* is an outlier. Figure 5—Supplementary Figure 1 is same as Figure 5a, but also highlights other drug resistance genes. Figure 5—Supplementary Figure 2a plots number of non-synonymous SNPs/kbp vs. protein conservation in Africa and SEA and show that *kelch13* follows the trend among genes in Africa, but has excess non-synonymous polymorphisms in SEA, while Figure 5—Supplementary Figure 2b plots number of synonymous SNPs/kbp vs. protein conservation in Africa and SEA and shows that k *elch13* follows the normal trend among genes in Africa, but has far fewer synonymous SNPs than expected in SEA. 

 Thus the authors conclude that high prevalence of non-synonymous SNPs in *kelch13* in SEA is not explainable by neutral evolution, but is consistent with neutral evolution in Africa. We would like to reemphasize that nowhere in the manuscript do the authors state or imply that strains from SEA have a higher N/S than strains from Africa.

- *Language used to describe the accrual of Nonsynonymous sites overstates the general trend of NS SNPs at conserved sites. Figure 4 shows that NS SNPs accrue at a higher rate in SEA across the set of conserved sites within Plasmodium and at the set of conserved sites across Eukaryotes. However, the language used to describe this figure in the legend states that “NS polymorphisms… in SEA are more likely to occur at conserved positions.” This statement implies that this is a general genome-wide trend of higher rate of fixing NS SNPs at sites with high conservation. Perhaps extending this legend to mention that NS SNPs are “are more likely to occur at conserved positions when compared to Africa” would avoid this implied generalization. Additionally, on page 3 the authors state “these mutations preferentially occur at the conserved sites”. This statement repeats the highlighted issue above, and adds the word “preferentially” which may create an unintentional teleological statement about the occurrences of NS mutations. NS SNPs occur at a higher rate within the pre-selected subset of conserved sites when compared to Africa, but the figures do not show a general genome-wide preference for NS mutations at conserved sites. *

**Author response: **We would like to thank the reviewer for pointing out this mistake. We have modified the Figure legend in the revised manuscript to “Non-synonymous polymorphisms specifically observed in SEA are more likely to occur at conserved positions when compared to non-synonymous polymorphisms specifically observed in Africa” as suggested. We have also modified the statement “This may be important for the acquisition of antimalarial drug resistance since these mutations preferentially occur at the conserved sites” to “This may be important for the acquisition of antimalarial drug resistance since drug-resistance mutations preferentially occur at the conserved sites”.

*Minor clarifications are as follows:*

- *“Kelch” is misspelled multiple times throughout the article.*
*  *
- *“Cambodia” is misspelled in multiple figures.*
*  *
- *“PNG” Abbreviation should be written out fully.*

 

**Author response: **Thanks for pointing out these mistakes; we have corrected these mistakes in the revised manuscript. We would be happy to address any further concerns that you may have about the revised version of the manuscript.

# Peer review: Referee response for version 1

**DOI:** 10.5256/f1000research.10094.r15669

**Published:** 2016-08-30

**Authors:** François H. Nosten

**Affiliations:** 1 Nuffield Department of Clinical Medicine, University of Oxford, Oxford, UK

In this paper the authors analyze the data produced by the MalariaGen community that provides high quality data on over 900,000 SNP in more than 3000 samples from 22 countries. The authors report a significantly higher ratio of non-synonymous to synonymous polymorphisms in *P. falciparum *from South-East Asia compared to Africa and propose that this could explain the emergence of resistance in Asia. However this observation (and also the higher multiplicity of infections in Africa) is not novel.

 Furthermore the large number of K13 alleles, their dynamics and relative contributions to the clinical phenotype are likely to also be important. I think they could improve by showing that their analysis helps to understand the spread of a specific allele of K13 such as the C580Y.

 It would be useful if the authors could provide more explanations as to why they believe their findings are innovative and how they help us to understand the evolution of artemisinin resistance, since the mechanism is clearly different to that of other antimalarials.

I have read this submission. I believe that I have an appropriate level of expertise to confirm that it is of an acceptable scientific standard, however I have significant reservations, as outlined above.

# Response

**Published:** 2016-10-17

**Authors:** Gajinder Singh

**Affiliations:** International Centre for Genetic Engineering and Biotechnology, New Delhi, India

Thank you for your review and comments.

*In this paper the authors analyze the data produced by the MalariaGen community that provides high quality data on over 900,000 SNP in more than 3000 samples from 22 countries. The authors report a significantly higher ratio of non-synonymous to synonymous polymorphisms in P. falciparum from South-East Asia compared to Africa and propose that this could explain the emergence of resistance in Asia. However this observation (and also the higher multiplicity of infections in Africa) is not novel.*

**Author response: **We are not aware of any manuscript that reported higher N/S ratio of *P. falciparum* of SEA strains of *P. falciparum* compared to African strains. If the reviewer is referring to the MalariaGen study (Elife. 2016), the authors in that manuscript did not report or suggest these results. In fact the authors of the MalariaGEN wrote that at the gene level “we found virtually identical distributions of the ratio of non-synonymous to synonymous mutations (N/S ratio) in the two regions”, however, no statistical test was performed by the authors. Furthermore, no comparison of N/S at the sample level was performed in the MalariaGEN study. We have added these statements to the Discussion section of the revised manuscript.

 We agree with the reviewer that the general conclusion of a higher multiplicity of infection in Africa is not novel, indeed we wrote “The rate of mixed infection is generally lower in areas of low-transmission such as SEA ^20^” before we reported our results. However we have confirmed the higher mixed infection rate in Africa compared to SEA in the MalariaGEN dataset, which is the largest whole-genome dataset on *P. falciparum* till date. We have added these statements to the Discussion section of the revised manuscript.

*Furthermore the large number of K13 alleles, their dynamics and relative contributions to the clinical phenotype are likely to also be important. I think they could improve by showing that their analysis helps to understand the spread of a specific allele of K13 such as the C580Y.*

**Author response: **Our manuscript is an attempt to understand why in general anti-malarial resistance often arises and spreads in SEA and it is beyond the scope of this manuscript to understand why a specific allele of K13 is dominating in certain parts of SEA.

*It would be useful if the authors could provide more explanations as to why they believe their findings are innovative and how they help us to understand the evolution of artemisinin resistance, since the mechanism is clearly different to that of other antimalarials.*

**Author response: **We have modified the Discussion section of the manuscript to clarify how our results differ from the MalariaGEN study. We have also discussed the implications of our results for the current wave of artemisinin resistance. These findings have not been previously reported. As stated previously our manuscript is an attempt to understand why in general anti-malarial resistance often arises and spreads in SEA, but whether and how the evolutionary mechanism of artemisinin resistance is different from other antimalarial is beyond the scope of this manuscript.

 We would again like to thank you for comments on our manuscript, and would be happy to address any further concerns that you may have about the revised version of the manuscript.
